# TGF-*β*1-Induced Expression of the Poor Prognosis SERPINE1/PAI-1 Gene Requires EGFR Signaling: A New Target for Anti-EGFR Therapy

**DOI:** 10.1155/2009/342391

**Published:** 2009-04-09

**Authors:** Rohan Samarakoon, Craig E. Higgins, Stephen P. Higgins, Paul J. Higgins

**Affiliations:** Center for Cell Biology and Cancer Research, Albany Medical College, 47 New Scotland Avenue, Albany, NY 12208, USA

## Abstract

Increased transforming growth factor-*β* (TGF-*β*) expression and epidermal growth factor receptor (EGFR) amplification accompany the emergence of highly aggressive human carcinomas. Cooperative signaling between these two growth factor/receptor systems promotes cell migration and synthesis of stromal remodeling factors (i.e., proteases, protease inhibitors) that, in turn, regulate tumor invasion, neo-angiogenesis and inflammation. ranscript profiling of transformed human cells revealed that genes encoding wound healing, matrix remodeling and cell cycle proteins (i.e., the “tissue repair” transcriptome) are significantly up-regulated early after growth factor stimulation. The major inhibitor of plasmin generation, plasminogen activator inhibitor-1 (PAI-1), is among the most highly induced transcripts during the phenotypic transition initiated by TGF-*β* maximal expression requires EGFR signaling. PAI-1 induction occurs early in the progression of incipient epidermal squamous cell carcinoma (SCC) and is a significant indicator of poor prognosis in epithelial malignancies. Mouse modeling and molecular genetic analysis of complex systems indicates that PAI-1 regulates the temporal/spatial control of pericellular proteolysis, promotes epithelial plasticity, inhibits capillary regression and facilitates stromal invasion. Defining TGF-*β*1-initiated signaling events that cooperate with an activated EGFR to impact the protease-protease inhibitor balance in the tumor microenvironment is critical to the development of novel therapies for the clinical management of human cancers.

## 1. Introduction

Transition of a normal epithelial cell to an early malignant phenotype often involves mutation of the p53 and p21^*ras*^ genes and progressive increases in autocrine TGF-*β*1 expression [1–10]. Elevated TGF-*β*1 production, in fact, typifies advanced pathologies in both mouse and human SCC [8, 10, 11]. Despite relatively high concentrations of TGF-*β* in the immediate tumor microenvironment, some malignant epithelial cells become refractory to TGF-*β*1-initiated proliferative arrest likely due to reductions in either TGF-*β*RII and/or SMAD4 levels as well as the now recognized p21^*ras*^-dependent antagonism of TGF-*β*1-mediated growth inhibition/apoptosis [10–13]. In certain epithelial malignancies, moreover, resistance to TGF-*β*1-mediated growth suppression is often coupled with EGFR amplification or dysregulated EGFR signaling, particularly during the later stages of tumor development [14–19]. The associated reprogramming of gene expression initiates and perpetuates TGF-*β*1-induced cellular “plasticity” (usually referred to as epithelial-to-mesenchymal transition or EMT) which facilites tumor invasion and metastasis [8, 20–25].

Microarray of the EMT transcriptome in several clinically relevant model systems has provided insights into the specific repertoire of “plasticity” genes. Plasminogen activator inhibitor type-1 (PAI-1; SERPINE1), the major physiologic regulator of the pericellular plasmin-generating cascade, is a prominent member of the subset of TGF-*β*1-induced, EMT-associated genes in human malignant keratinocytes [21, 26, 27]. In epithelial cells undergoing a mesenchymal-like conversion in response to the E-cadherin transcriptional repressors Snail, Slug or E47, PAI-1 upregulation appears to be an essential characteristic of the plastic phenotype [28]. The association between PAI-1 expression and tumor “progression” has significant clinical implications. Current data suggest that this serine protease inhibitor maintains an angiogenic “scaffold,” stabilizes nascent capillary vessel structure, and facilitates tumor cell invasion through precise control of the peritumor proteolytic microenvironment [29–31]. Increased PAI-1 expression is, in fact, an early event in the progression of epidermal SCC, often localizing to tumor cells and myofibroblasts at the invasive front [24, 32–36] and, most importantly, is a biomarker with significant prognostic value [37]. Indeed, two of the best-validated prognostic indicators (level of evidence [LOE] = 1) in breast carcinoma are the serine protease urokinase plasminogen activator (uPA) and its endogenous inhibitor PAI-1 [38]. Certain PAI-1 tumor thresholds predict both poor prognosis and reduced disease-free survival in patients with breast, lung, ovarian, and oral SCC [29, 38] with the expression amplitude frequently associated with the 4G polymorphism at the PE1 E box motif in the PAI-1 promoter [37]. Identification of PAI-1 in tumor-proximal stromal myofibroblasts, furthermore, implies a more global involvement in modulating cellular invasive potential [34–36], perhaps as a matricellular effector of epithelial motility [39], invasion and the associated angiogenic response [24, 30, 31, 40, 41].

Recent findings clearly implicate EGFR/MEK/*rho*-ROCK signaling as required for PAI-1 expression in TGF-*β*1-stimulated cells. E box motifs (CACGTG) in the PAI-1 PE1/PE2 promoter regions, moreover, are platforms for a MAP kinase-directed upstream stimulatory factor (USF) subtype switch (USF-1 → USF-2) in response to growth factor addition [42–44] suggesting that the EGFR/MEK/*rho*-ROCK axis impacts PAI-1 expression through USF-dependent transcriptional controls. The continued definition of TGF-*β*1-activated pathways that influence expression of this important target gene may lead to therapeutically useful approaches to manage human cancer. This paper, therefore, reviews data regarding the rapid transactivation of the EGFR in TGF-*β*1-stimulated cells suggesting cooperativity between TGF-*β*1 and EGFR → MAP kinase pathways in PAI-1 gene expression.

## 2. EGFR Signaling Is Required for TGF-*β*1-Induced PAI-1 Expression

TGF-*β*1 mobilizes both SMAD-dependent and -independent signaling [45] although the individual roles of specific cross-pathway events on PAI-1 expression are not well understood. Several recent studies demonstrated that TGF-*β*1-induced rapid EGFR transactivation highlighting cooperativity between TGF-*β*1 and EGFR signaling events in vascular, epithelial, and endothelial cells. Indeed, PAI-1 induction in response to TGF-*β*1 is significantly attenuated by an EGFR pharmacologic inhibitor (AG1478), by molecular targeting of EGFR activity (i.e., by adenoviral delivery of EGFR^Y721A^ kinase-dead constructs) and, more importantly, by genetic ablation of the EGFR in mouse fibroblasts [43, 46, 47] with PAI-1 “rescue” evident in EGFR^−/−^ cells engineered to express an EGFR construct. TGF-*β*1 treatment, moreover, specifically increased EGFR phosphorylation at the Y845 *src*-target residue; either mutation of this residue (EGFR^Y845F^) or transfection of a DN pp60^c-*src*^ construct completely blocked TGF-*β*1-dependent PAI-1 induction. Similarly, TGF-*β*1 failed to stimulate PAI-1 expression in cultured mouse embryonic fibroblasts (MEFs) genetically deficient in three *src* family kinases (i.e., c-*src*, c-*yes*-, c-*fyn*- null fibroblasts; SYF^−/−/−^) compared to identically stimulated wild-type SYF^+/+/+^ cells. PAI-1 synthesis was restored in SYF^−/−/−^ MEFs engineered to re-express a wild-type pp60^c-*src*^ [47] providing proof-of-principle for involvement of this particular *src* kinase in the inductive response. The highly specific *src* family kinase inhibitor SU6656, morevover, effectively blocked TGF-*β*1-initiated increases in both pp60^c-*src*^ and EGFR phosphorylation as well as pp60^c-*src*^ and EGFR activation (at the Y416 and Y845 residues, resp.). pEGFR^Y845^ phosphorylation in response to TGF-*β*1 was evident, furthermore, in wild type but not SYF^−/−/−^ fibroblasts. The TGF-*β*1-dependent formation of EGFR/pp60^c-*src*^ complexes [46] and EGFR^Y845^ phosphorylation and the inhibition of TGF-*β*1- (but not PDGF-) induced PAI-1 expression by the EGFR^Y845F^ mutant as well as a DN-Src construct [47] collectively implicate EGFR/pp60^c-*src*^ interactions and, in particular, the EGFR^Y845^ pp60^c-*src*^ site in the kinase domain activation loop in signal propagation [48]. The time course of TGF-*β*1-initiated SMAD2/3 activation, in contrast, was similar in both wild type and SYF^−/−/−^ MEFs confirming that, in the context of either EGFR or *src* family kinase deficiency, SMAD2/3 activation occurs but is not sufficient for PAI-1 induction. TGF-*β*1 stimulated ERK1/2 phosphorylation in EGFR^+/+^ but not in EGFR^−/−^ cells consistent with prior observations that TGF-*β*1-dependent ERK1/2 activation is downstream of EGFR signaling [43, 46]. EGFR^−/−^ MEFs, however, are fully capable of responding to exogenous TGF-*β*1 as SMAD2 was effectively activated (i.e., phosphorylated) in both wild type and EGFR^−/−^ fibroblasts [47].

## 3. The PAI-1 Gene Is a Model of TGF-*β*1-Initiated Cooperative EGFR Signaling

While TGF-*β*1 receptors phosphorylate SMADs downstream of growth factor engagement, it appears that the Rho/ROCK pathway modulates the duration of SMAD2/3 phosphorylation [47]. How Rho/ROCK impact TGF-*β*1-initiated SMAD2/3 activation and subcellular localization [49, 50] is not known but this pathway may function to provide efficient SMAD2/3 activation for extended periods. Alternatively, Rho/ROCK signaling may be required to inhibit negative regulation of SMAD2/3 function by inactivation of SMAD phosphatases sustaining, thereby, SMAD2/3 transcriptional actions (e.g., [51, 52]). TGF-*β*1-induced SMAD2 phosphorylation is not altered by EGFR blockade either pharmacologically (with AG1478), molecularly (by expression of EGFR^Y721A^ or EGFR^Y845F^), or by the genetic absence of EGFR [47]. Clearly, while SMAD2/3 activation may be necessary it is not sufficient for TGF-*β*1-stimulated PAI-1 expression in the absence of EGFR signaling.

**Figure 1:**
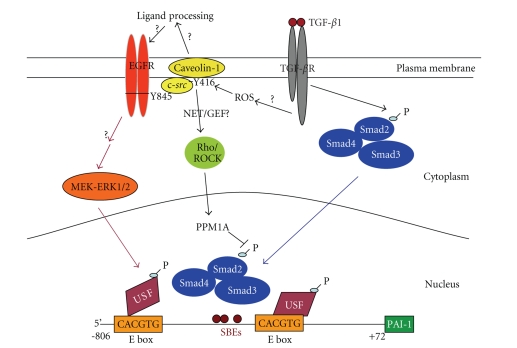
Model for TGF-*β*1-induced PAI-1 expression. TGF-*β*1 activates two distinct signaling pathways to initiate PAI-1 transcription. Rho/ROCK are required to maintain SMAD phosphorylation and ERK activation (through to be defined mechanisms) while the pp60^c-*src*^-activated EGFR (at the Y845 site) signals to MEK-ERK initiating ERK/USF interactions resulting in USF phosphorylation and a subtype (USF-1 → USF-2) switch (e.g., [44]) at the PAI-1 PE1/PE2 E box sites. Collectively, these promoter-level events stimulate high level PAI-1 expression in response to TGF-*β*R occupancy. The actual mechanism underlying EGFR activation in response to TGF-*β*1 may involve direct recruitment of *src* kinases to the EGFR or the processing/release of a membrane-anchored EGFR ligand (e.g., HB-EGF). Events associated with TGF-*β*1 stimulation of the RhoA/ROCK pathway are similarly unclear. Rho/ROCK may regulate the activity and/or function of the SMAD phosphatase PPM1A impacting, thereby, the duration of SMAD-dependent transcription of target genes such as PAI-1. (modified from [47]).

It is apparent, therefore, that TGF-*β*1 stimulates PAI-1 expression through two distinct but cooperating pathways that involve EGFR/pp60^c-*src*^ → MEK/ERK signaling and EGFR-independent, but Rho/ROCK-modulated, TGF-*β*R-directed SMAD and ERK activation [47]. Interference with any of the specific individual elements in this dual cascade (EGFR/pp60^c-*src*^/MEK or Rho/p160ROCK) markedly reduced, and in some cases, completely inhibited PAI-1 expression. One model consistent with the available data [24, 40, 43, 44, 47, 53] suggests that SMADs and specific MAP kinase-targeted bHLH-LZ factors (such as USF) occupy their separate binding motifs at the critical TGF-*β*1-responsive PE2 region E box in the PAI-1 promoter (Figure 1). Dominant-negative interference with USF DNA-binding ability significantly reduced TGF-*β*1-mediated PAI-1 transcription [43, 44, 53]. Since MAP kinases regulate the DNA-binding and transcriptional activites of USF [40, 43], TGF-*β*R signaling through SMAD2/3 may actually cooperate with EGFR/MEK-ERK-activated USF to attain high level PAI-1 expression [40, 47]. SMADs are known to interact with E box-binding HLH-LZ factors such as TFE3 at the PE2 site in the PAI-1 geneat least in one cell type [54]. There is evidence, in fact, to suggest that such interacting complexes impact PAI-1 gene control since USF occupancy of the PAI-1 PE2 region E box site, which is juxtaposed to three SMAD-recognition elements, modulates transcription in response to TGF-*β*1 or serum [40, 43, 44, 53]. Current data indicate that recruitment of this multicomponent complex likely requires participation of the TGF-*β*1-stimulated EGFR → MEK/ERK and Rho/ROCK pathways for the optimal response of the PAI-1 gene to TGF-*β*1.

The mechanism of MAP kinase activation in TGF-*β*1-stimulated cells is just becoming clear. Upon ligand binding, the TGF-*β*RII undergoes autophosphorylation on three tyrosines (Y259, Y336, Y424), while Y284 is a target site for *src* kinases [55]. TGF-*β*RI is also subject to tyrosine phosphorylation postreceptor accupancy [56]. Such phosphorylated tyrosine residues provide docking sites for recruitment of Grb2/Shc/SOS complexes with subsequent mobilization of the *ras*-*raf*-MEK-ERK cascade [46, 47, 55]. Although ERKs are prominently activated in response to TGF-*β*1 [40, 43], perhaps the JNK and p38 MAP kinase pathways are better characterized targets of TGF-*β*1-initiated signaling. TGF-*β*1 rapidly activates JNK through MKK4 and p38 via MKK3/6 perhaps even in a cell type-specific fashion contributing to the mechanistic complexity of pathway cross-talk. Each of these kinase systems, moreover, has been implicated in a cell type-dependency of PAI-1 gene control [40, 43, 55]. Should such pathways prove uniquely or, at least, preferentially utilized in specific cellular lineages, they may provide tumor type-specific targets for intervention therapy.

## 4. EGFR as a Potential Therapeutic Target for Regulating PAI-1 Expression

Modulation of EGFR/HER1 signaling by specific receptor function (kinase domain) inhibitors or neutralizing antibodies against specific EGFR1 ligands (e.g., HB-EGF antibodies) can be an attractive therapeutic modality (particularly in the context of neoplastic diseases associated with elevated TGF-*β*1 levels). This strategy would likely impact not only PAI-1 suppression but has the potential to regulate other proinvasive target genes. There is, in fact, increasing evidence that TGF-*β*1-induced connective tissue growth factor and fibronectin expression similarly involve EGFR/HER1 cooperative pathways (Samarakoon and Higgins, unpublished data). Moreover, PAI-1 repression by EGFR signaling blockade may also suppress tumor angiogenesis consistent with the well-established role of PAI-1 as an inhibitor of endothelial apoptosis and neovessel regression [40]. Combinatorial targeting of PAI-1 function using established small molecule PAI-1 inhibitors and genetic-based PAI-1 expression attenuation [40] coupled with disruption of EGFR signaling (e.g., with cetuximab or erlotinib) may impact, therefore, both cancer invasion and the associated angiogenic response, particularly in the context of a TGF-*β*1-rich tumor microenvironment.
